# Tumor Microenvironment: Insights from Multiparametric MRI in Pancreatic Ductal Adenocarcinoma

**DOI:** 10.3390/cancers18020273

**Published:** 2026-01-15

**Authors:** Ramesh Paudyal, James Russell, H. Carl Lekaye, Joseph O. Deasy, John L. Humm, Muhammad Awais, Saad Nadeem, Richard K. G. Do, Eileen M. O’Reilly, Lawrence H. Schwartz, Amita Shukla-Dave

**Affiliations:** 1 Department of Medical Physics, Memorial Sloan Kettering Cancer Center, New York, NY 10065, USA; 2 Department of Radiology, Memorial Sloan Kettering Cancer Center, New York, NY 10065, USA; 3 Department of Medicine, Memorial Sloan Kettering Cancer Center, New York, NY 10065, USA

**Keywords:** apparent diffusion coefficient, hoechst, hematoxylin and eosin, pancreatic ductal carcinoma, stroma, tumor microenvironment, volume transfer constant

## Abstract

**Background/Objectives:** The tumor microenvironment (TME) of pancreatic ductal adenocarcinoma (PDAC) is characterized by an enriched stroma, hampering the effectiveness of therapy. This co-clinical study aimed to (1) provide insight into early post-treatment changes in the TME using multiparametric magnetic resonance imaging (mpMRI)-derived quantitative imaging biomarkers (QIBs) in a preclinical PDAC model treated with radiotherapy and correlate these QIBs with histology; (2) evaluate the feasibility of obtaining these QIBs in patients with PDAC using clinically approved mpMRI data acquisitions. **Methods**: Athymic mice (*n* = 12) at pre- and post-treatment as well as patients with PDAC (n = 11) at pre-treatment underwent mpMRI including diffusion-weighted (DW) and dynamic contrast-enhanced (DCE) data acquisition sequences. DW and DCE data were analyzed using monoexponential and extended Tofts models, respectively. DeepLIIF quantified the total percentage (%) of tumor cells in hematoxylin and eosin (H&E)-stained tissues from athymic mice. Spearman correlation and Wilcoxon signed rank tests were performed for statistical analysis. **Results**: In the preclinical PDAC model, mean pre- and post-treatment ADC and K^trans^ values differed significantly (*p* < 0.01), changing by 20.50% and 20.41%, respectively, and the median total tumor cells quantified by DeepLIIF was 24% (range: 15–53%). Post-treatment ADC values and relative change in v_e_ (rΔv_e_) showed a significant negative correlation with total tumor cells (*ρ* = −0.77, *p* < 0.014 for ADC and *ρ* = −0.77, *p* = 0.009 for rΔv_e_). In patients with PDAC, pre-treatment mean ADC and K^trans^ values were 1.76 × 10^−3^ (mm^2^/s) and 0.24 (min^−1^), respectively. **Conclusions**: QIBs in both preclinical and clinical settings underscore their potential for future co-clinical research to evaluate emerging drug combinations targeting both tumor and stroma.

## Simple Summary

Pancreatic ductal adenocarcinoma (PDAC) is characterized by a highly heterogeneous tumor microenvironment (TME), enriched with stromal components such as cancer-associated fibroblasts and dense extracellular matrix, which contribute to therapeutic resistance. Multiparametric magnetic resonance imaging (mpMRI) can yield valuable quantitative imaging biomarkers (QIBs) derived from diffusion-weighted (DW) and dynamic contrast–enhanced (DCE) MRI that can be used to assess characteristics of the TME such as cellularity and vascular permeability. Meanwhile, histological staining (Hoechst, hematoxylin and eosin [H&E]) provides insights into the TME spatial organization. Harnessing mpMRI and histology together in PDAC is vital for combating therapeutic resistance and enhancing treatment efficacy. This study establishes a foundation for future co-clinical research to evaluate emerging drug combinations that target both tumor and stroma, thus advancing our understanding of the TME in PDAC.

## 1. Introduction

Despite advances in multimodal therapeutic strategies, pancreatic ductal adenocarcinoma (PDAC), currently the fourth leading cause of cancer-related deaths in the United States [1], remains largely refractory to therapy, owing to its aggressive tumor biology [2]. Comprising immune cells, fibroblasts, blood vessels, signaling molecules, and extracellular matrix, PDAC is characterized by stromal desmoplasia and vascular dysfunction within the tumor microenvironment (TME) [3]. The stroma acts as a physical and biochemical barrier to drug delivery. PDAC TME remains poorly understood, underscoring the need for mechanism-driven therapies in patients with PDAC [4,5]. Correspondingly, there is a critical demand for noninvasive biomarkers that can uncover the biological mechanisms driving tumor development, which not only supports more effective disease monitoring but also informs for the development of personalized therapeutic approaches [6]. Quantitative imaging biomarkers (QIBs) derived from multiparametric magnetic resonance imaging (mpMRI), including diffusion-weighted (DW) and dynamic contrast-enhanced (DCE) MRI data acquisition sequences, provide characteristics of tumor physiology, such as cellularity, perfusion, and vascular permeability [7,8], showing great promise in the evaluation of the local TME [9,10,11] and supporting the assessment of treatment response in both preclinical and clinical studies of PDAC [12,13,14,15,16].

High tumor cellularity hinders and restricts water diffusion, resulting in lower apparent diffusion coefficient (ADC) values [15]. Moreover, advanced diffusion kurtosis imaging (DKI) extends beyond ADC measurement by quantifying non-Gaussian water diffusion, thereby offering insights into tumor tissue microstructure [17]. The DCE data modeled with an extended Tofts model allows us to estimate the vascular permeability, and volume fractions of the extravascular extracellular space (EES) and blood plasma [18], enabling early treatment response assessment in preclinical and clinical PDAC studies [12,13,14,16,19,20]. Together, these parameters capture tumor features, dense stroma, and aberrant vasculature, promising to aid the development of new therapeutic strategies for PDAC [21]. Notably, in both preclinical models and clinical studies, mpMRI-derived QIBs have shown promise in evaluating changes to the TME induced by stromal-targeted therapies that aim to disrupt stromal components, reduce interstitial fluid pressure, and thereby enhance drug delivery and therapeutic efficacy [12,13,19,22,23,24,25]. Additionally, mpMRI-derived QIBs have shown significant association with tumor response to chemotherapy and radiotherapy in patients with PDAC [14,16,19,20,26].

Understanding the architecture of the TME in PDAC is important for developing novel drugs and therapeutic strategies as well as assessing their effectiveness in overcoming treatment resistance [27]. High-resolution histological evaluation of tumor sections with Hoechst and hematoxylin and eosin (H&E) staining reveals insights into the TME by uncovering perfusion and morphological characteristics [28]. Various studies have examined the correlation between QIBs, including those reflecting tissue cellularity, vascularity, and microstructure, with histological measurements of tumor tissue, such as tumor cell count [10,22,29,30,31,32]. Barnes et al. [30] reported that in breast cancer xenografts in athymic mice, median central-slice ADC values showed a significant positive correlation with extracellular space measured by H&E staining. Kalber et al. [28] used H&E staining to identify necrotic regions and Hoechst 33,342 uptake to indicate perfused vasculature in LS174T colorectal liver metastases in nude mice [29]. Klaassen et al. [31] found that ADC, volume transfer constant (K^trans^), and volume fraction of EES (v_e_) values were significantly correlated with the collagen fraction in patients with PDAC. Farace et al. [33] demonstrated that contrast agent (CA) distribution correlates with stromal content, especially in tumor peripheries showing early enhancement, suggesting regions of high stromal density. Mayer et al. [32] employed DKI to assess tissue microstructure, enabling characterization of tumor and stromal components in PDAC.

There is a critical unmet need to characterize TME in PDAC using mpMRI-derived QIBs, particularly for developing and evaluating novel stroma-directed therapeutic strategies [34,35]. To bridge the translational gap in PDAC treatment, researchers are increasingly using animal models that closely replicate human tumor biology [36]. The present co-clinical study has two aims: (1) to provide insight into early post-treatment changes in the TME using mpMRI-derived QIBs in a preclinical model of PDAC treated with radiotherapy and to correlate these QIBs with histology, and (2) to evaluate the feasibility of obtaining these QIBs in patients with PDAC using clinically approved mpMRI data acquisition sequences at pre-treatment. This study establishes a foundation for future co-clinical research to evaluate emerging drug combinations that target both the tumor and the stroma, thereby advancing our understanding of the TME in PDAC.

## 2. Materials and Methods

### 2.1. Preclinical Study with Animals and Tumor Models

The Institutional Animal Care and Use Committee of our center approved all animal procedures. Animals were maintained in temperature- and humidity-controlled rooms, and food and water were provided ad libitum. Animals were housed for a minimum of two weeks prior to mpMRI acquisition. Tumor cells were generously provided by Professor RH Vonderheide (University of Pennsylvania) and were originally derived from a murine pancreatic tumor, genotype Pdx1-Cre; LSL KRASG12D; Trp53R172H/wt [37]. A total of 2 × 10^5^ KPC 4662 tumor cells were subcutaneously injected into the right shoulder region of thirteen athymic mice (*n* = 13), allowing simultaneous visualization of both the tumor and the heart, which enabled extraction of the signals required for pharmacokinetic modeling of tumor tissue and the arterial input function (AIF), as described by Zhou et al. [38]. Daily monitoring was conducted to track weight loss and any symptoms. Tumors were allowed to grow for at least 10 days. Pre-treatment mpMRI was performed 10–15 days after tumor inoculation, and post-treatment mpMRI was performed the next day (within 20 h) after irradiation.

### 2.2. Preclinical MRI Data Acquisition

Mice were prepared for MRI acquisition by inducing general anesthesia with 1.5% isoflurane in 70% N_2_/30% O_2_ delivered through a nose cone. A tail vein catheter, along with extension tubing long enough to extend from the magnet isocenter to the end of the magnet bore, was placed. The tubing was preloaded with gadolinium-based CA (details below) to reduce dead volume effects. Each mouse’s core temperature was maintained at 37  ±  1  °C in the magnet bore, with heating controlled with a thermal regulator system (SA Instruments, Stony Brook, NY, USA). The respiration rate and core body temperature were continuously monitored throughout the MRI procedure.

mpMRI data acquisitions were performed using a horizontal small-animal 7-T positron emission tomography/magnetic resonance imaging (PET/MRI) scanner (BioSpec 70/30; Bruker BioSpin MRI GmbH, Ettlingen, Germany, running ParaVision 5.1). T_2_-weighted (w) data acquisition was first performed, followed by DW and DCE MRI as well as post-T_1_w.

T_2_w images were acquired using a 2D fast spin-echo RARE (Rapid Acquisition with Relaxation Enhancement) sequence with the following parameters: repetition time (TR) = 3254.13 ms, echo time (TE) = 50.428 ms, slice thickness = 1 mm, slice spacing = 1.1 mm, number of averages (NA) = 2, number of slices (NS) = 30, acquisition matrix size (MS): 160 × 192, leading to pixel size = 0.188 × 0.169 mm^2^ and field of view (FOV) = 30 mm.

DW images were acquired with four b-values (0, 100, 400, and 700 s/mm^2^) with TR/TE = 1700/25.46 ms, MS = 80 × 120, NA = 2, NS = 6, pixel size = 0.263 × 0.249 mm^2^, slice thickness = 1.0 mm, and slice spacing = 1.1 mm.

In DCE MRI, T_1_w dynamic images were acquired using a FLASH (Fast Low Angle Shot) sequence with the following parameters: TR/TE = 52.0/3.12 ms, NA = 2, NS = 6, flip angle (FA) = 15°, MS = 132 × 106, and pixel size = 0.265 × 0.278 mm^2^. After acquiring ~20 precontrast images, 100 μL of a gadolinium-based CA (Magnevist, Bayer Healthcare, Wayne, NJ, USA), along with 20 μL of saline flush, was injected at a constant rate via a tail vein catheter. The acquisition time for each phase was approximately 6 s. For T_10_ mapping, T_1_w images were acquired using the same parameters at four TR values (100, 200, 800, and 2000 ms). Both pre- and post-treatment mpMRI data were acquired from twelve athymic mice (*n* = 12).

All acquired MRI data were exported in the DICOM format, and DICOM images were converted to the NIFTI format for image analysis [39].

### 2.3. Irradiation

Tumor-bearing mice (n = 12) were locally irradiated with a radiation dose of 10 Gy administered using a dedicated small-animal radiotherapy device (Precision X-Ray, Madison, CT, USA) [40] after mpMRI. Radiation was delivered using a photon beam (225 kV, 13 mA, 3 mm Cu) with a dose rate of approximately 3 Gy/min using a 10 mm diameter collimator. Mice received continuous isoflurane gas anesthesia (2% isoflurane, 1 L/min in air).

### 2.4. Histology

Following irradiation and post-treatment MRI, tumor-bearing mice were injected intravenously with 0.1 mL Hoechst 3342 (10 mg/mL in saline; Millipore Sigma, St. Louis, MO, USA) and euthanized using CO_2_ inhalation 1 min later. Tumors were removed, frozen in optimal cutting temperature, and sectioned at a thickness of 10 μm. It was not possible to reliably cut tumors in the plane of the MR images; to compensate, each tumor was sectioned throughout its entire depth, 2–3 sections being obtained every 200 μm. Unfixed sections were imaged using an Olympus microscope, where blue fluorescence captures the vasculature, indicating the presence of perfused vessels. Sections from mice (*n* = 10 mice) were subsequently stained with H&E (Vector Labs, Burlington, CA, USA) and analyzed using DeepLIIF, as detailed below. Of thirteen mice, one died following the mpMRI scan. Among the remaining twelve mice that underwent treatment and had complete pre- and post-treatment mpMRI data, tumor tissue could not be obtained from two mice that died after the scans.

### 2.5. DeepLIIF

DeepLIIF, (v1.0, GitHub), is a custom Python-based deep-learning, cloud-native platform framework designed for processing H&E images [41]. Histological images from ten athymic mice (*n* = 10) were first preprocessed to enable efficient downstream analysis. Large images were automatically assessed for orientation and rotated to maintain a consistent horizontal layout when necessary. Each image was then subdivided into fixed-size tiles (4096 × 4096 pixels) by iteratively cropping row–column regions across the slide, and all tiles were stored in a dedicated output directory. Comprehensive metadata, including tile name, spatial coordinates, and dimensions, were generated in tabular format to preserve the spatial relationship of each tile relative to the original image. The resulting H&E tiles were subsequently processed using DeepLIIF. The processed outputs for each tile were reconstructed into stitched composite images, allowing visualization of the entire slide in a unified format. Quantitative data from each H&E tile, including the total number of tumor cells, nuclei, and the percentage of tumor cells, were aggregated across all tiles to report as mean and standard deviation (SD) values.

### 2.6. Clinical Study with Patients with PDAC

Our institutional review board approved this prospective study which was compliant with the Health Insurance Portability and Accountability Act. Patients with PDAC (*n* = 11, F/M = 3/8, median age = 58 (34–82) underwent mpMRI using a 3T GE MRI scanner (GE HealthCare, Waukesha, WI, USA) prior to treatment with systemic chemotherapy (mFOLFIRINOX; 5-Fluoruracil, leucovorin, irinotecan and oxaliplatin) and pancreatic enzyme replacement therapy (PERT, Pancrelipase (Pertzye)). Inclusion criteria for the study required a histopathologic or cytologic diagnosis of PDAC. Eligible patients had de novo or recurrent, previously untreated stage IV disease and were planning to receive treatment at our institution. Additional requirements included an ECOG performance status of 0–2, no PERT within the prior two weeks, and biliary stenting completed or planned before treatment if obstruction was present. Patients also needed to provide informed consent, be able to swallow oral capsules, be at least 18 years of age, and have an anticipated life expectancy of six months or longer. The intervention was as follows: participants took blinded, weight-based Pancrelipase with the first bite of every meal or snack for 20 weeks from enrollment, documenting intake and food consumption in study diaries. Evaluations occurred every 4 weeks alongside standard care visits. mFOLFIRINOX was administered every 2 weeks per institutional guidelines, with premedication, supportive care, and dose modifications as needed.

### 2.7. Clinical MRI Data Acquisition

mpMRI data acquisitions were performed using clinically approved standard MR sequences. T2w and pre- and post-contrast T1w images were acquired under breath-hold, followed by DW and DCE MRI on a GE scanner with a 30-element anterior (30AA) and 40-element posterior (40PA) receive coil configuration.

T_2_w-images were acquired using a fat-suppressed, fast spin-echo sequence with the following parameters: TR/TE = 4000/104.52 ms, NA = 1, MS = 512 × 224, slice thickness = 7 mm, FOV = 30–35 cm, and NS = 35–40.

Pre- and postcontrast 3D T_1_w MRI LAVA (Liver Acquisition with Volume Acceleration) was performed with the following parameters: TR/TE = 4.296/1.992 ms, NA = 1, matrix size = 320 × 224, slice thickness = 5 mm, and NS = 35–40.

Reduced field of view (rFOV) DW MRI [42] was performed with the following parameters: TR/TE = 4000/62.1 ms, MS = 160 × 80, slice thickness = 4 mm, NS = 30–35, b = 0, 500, 800 s/mm^2^, and NA = 2–4. The acquisition time for rFOV DW MRI was ≤4 min.

T_1_w dynamic images were acquired using the Differential Subsampling with Cartesian ordering (DISCO) and Stack-of-stars (STAR) for Temporal and Respiratory motion management sequence [43]. DISCO-STAR integrates pseudo-random k-space sampling with radial acquisition in the xy-plane to improve motion robustness, combined with Cartesian sampling along the slice (z) direction. This enables high-quality dynamic imaging, especially for motion-prone areas like the abdomen. DCE images were acquired with the following parameters: FA = 12°, TR/TE = 4.296/1.992 ms, MS = 320 × 224 (reconstructed to 256 × 256 by zero-filling), FOV = 30–35 cm, slice thickness = 5 mm (covering the liver and pancreas), NS = 25–30, and 15 phases with temporal resolution of 10 s per phase. After acquiring 3–4 precontrast images, a bolus of 0.1 mmol/kg gadolinium-based CA (Gadovist^®^; Bayer Healthcare, Leverkusen, Germany) was delivered through an antecubital vein catheter at 2 cc/s, followed by a 20-to 40 mL saline flush delivered through an MR-compatible programmable power injector (Spectris, Medrad, Indianola, PA, USA). The acquisition time for DISCO-STAR was ≤5 min. T_10_ mapping was performed using a LAVA sequence and a variable flip angle technique at flip angles of 5°, 15°, and 30°, as described by Do et al. [16]. For the clinical data, all images were acquired using standard diagnostic imaging protocols, and as a result, additional B1 mapping data were not available.

### 2.8. DW- and DCE-MRI Data Modeling and Analysis

**Figure 1:**
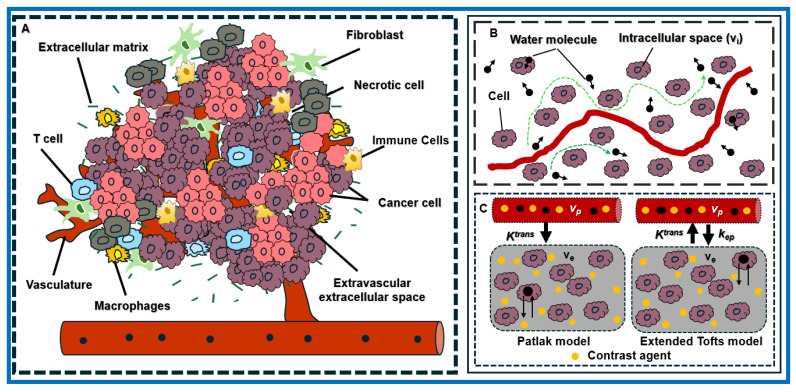
(**A**) Schematic representation of the PDAC TME, highlighting cancer cells, immune cells, fibroblasts, vasculature, and the EES. (**B**) Illustration of the Brownian motion of water molecules within tumor tissue, where cells impede water diffusion. (**C**) Workflow of two pharmacokinetic models, Patlak and the extended Tofts, used in DCE data modeling. CA extravasation from blood plasma into the EES is characterized by K^trans^. v_e_ and v_p_ represent the volume fraction of the EES and plasma space, respectively. k_ep_ represents the transport rate constant for CA from the EES back into blood plasma.

Figure 1 shows a representative dense and heterogeneous TME in PDAC, comprising cancer cells, fibroblasts, and abnormal vasculature that drive tumor progression, contribute to therapeutic resistance, and modulate the MR signal. DW images capture restricted water diffusion due to dense tumor tissue cells. Meanwhile, DCE images reflect signal enhancement resulting from CA extravasation across the capillary wall. The MR-based physiological models used for mpMRI data analysis are described below.

A monoexponential model for DW signal attenuation against b-value, is given by

(1) $$S_{b}=S_{b}\;e^{-b\times ADC}$$

where S_b_ and S_0_ are the signals with and without the diffusion gradient parameter, b (s/mm^2^), reflecting the strength and timing of the diffusion gradients applied during DW MRI, and ADC (mm^2^/s) represents the water diffusivity in tissue.

In DCE MRI, the longitudinal relaxation rate (R_1_ = 1/T_1_) of tumor tissue water protons is calculated from the signal intensity before, during, and after CA administration [18]. R_1_ (t) is assumed to be linearly related to tissue CA concentration, C_t_(t), under the fast water exchange limit approximation:

(2) $$R_{1t}(t)=R_{10}+\;r_{1}\;C_{t}\left(t\right)⟶{\;∆R}_{1t}(t)={\;R}_{1t}(t)-R_{10}=\;r_{1}\;C_{t}(t)$$

where R_1t_(t) is the time course of the R_1_, R_10_ is the intrinsic R_1_ of the water protons. The longitudinal relaxivities (r_1_) used for preclinical studies at 7 T and clinical studies at 3 T were 4.0 mM^−1^·s^−1^ (Magnevist) [44] and 5.0 mM^−1^·s^−1^ (Gadovist) [45], respectively.

The two-parameter Patlak model uses a linear graphical approach to estimate two parameters, K^trans^ and v_p_, after CA administration, and the equivalent observation equation is given as follows [46,47]:

(3) $$C_{t}\left(t\right)=K^{trans}\int_{o}^{t}C_{p}\left(\tau \right)d\tau +v_{p}C_{p}\to \;\frac{{\left(1-HCt\right)∆R}_{1t}\left(t\right)}{{∆R}_{1a}\left(t\right)}=K^{trans}\frac{\int_{o}^{t}{∆R}_{1a}\left(\tau \right)d\tau }{{∆R}_{1a}\left(t\right)}+v_{p}$$

where K^trans^ and v_p_ are associated with the tumor vascularity, and C_p_(t) is the time course of plasma CA concentration (called [AIF]). ΔR_1a_ and ΔR_1t_ refer to the change in R_1_ at arterial blood and tissue, respectively, and Hct is the hematocrit level at the microvasculature (Hct =0.45).

The three-parameter extended Tofts model describes CA extravasation into the tissue voxel as a function of time, based on the reversible exchange between the blood plasma and the EES as follows [18]:

(4) $$C_{t}\left(t\right)=K^{trans}\int_{o}^{t}e^{-k_{ep}(t-\tau \;)}C_{p}\left(\tau \right)d\tau \;+v_{p}C_{p}\;$$

The equivalent observable equation is given by

(5) $$\;{(1-HCt)∆R}_{1t}\left(t\right)=K^{trans}\int_{o}^{t}e^{-k_{ep}(t-\tau \;)}{∆R}_{1a}\left(\tau \right)d\tau +v_{p}∆R_{1a}$$

where k_ep_ = K^trans^/v_e_ is the transfer rate constant from the EES to the vascular space, and v_e_ is the volume fraction of the EES.

### 2.9. Image Processing and Data Analysis

#### 2.9.1. Image Processing and Data Analysis for the Preclinical PDAC Model

Using ITK-SNAP (v3.6), regions of interest (ROIs) were manually contoured on DW (b = 0 s/mm^2^) and DCE images, referencing high b-value DW images and late-phase DCE images. DW and DCE image processing, along with QIB parametric map generation and the extraction of QIB values, were performed using an in-house software, MRI-QAMPER (MRI Quantitative Analysis Multi-Parametric Evaluation Routines), that supports T_10_ mapping [39]. The AIF was extracted according to previously published method, as detailed previously by Zhou et al. [38], using the automated detection algorithms implemented in MRI-QAMPER software (v2). Voxel-wise kinetic modeling was performed using a nonlinear least-squares algorithm. Parameter bounds were applied to constrain fits within physiologically reasonable ranges (K^trans^ [0, 2.0], v_e_ [0, 1.0], and v_p_ [0, 0.5]). Voxels were excluded if the fitting procedure failed to converge, returned unphysical parameter values, or exhibited poor goodness-of-fit, ensuring robust and reliable QIBs values and maps. The relative percentage change in QIB values between pre- and post-treatment was calculated as follows:

(6) $$∆X(\%)=\frac{\left|X_{post-Tx}-X_{pre-Tx}\right|}{X_{pre-Tx}}\times 100\;$$

where X refers to the following QIB values: ADC, K^trans^, v_e_, and v_p_.

The changes in pre- and post-Tx ADC, K^trans^, v_e_, and v_p_ values between pre- and post-treatment were compared using the Wilcoxon signed rank test (WSRT), with effect sizes (r) with corresponding 95% confidence intervals (CIs) included for all comparisons in for the preclinical model of PDAC model. Owing to the exploratory nature of this feasibility study, we did not perform multiple-comparison adjustments. Associations between QIBs and quantitative features values obtained from DeepLIIF-processed H&E staining were assessed using Spearman correlation. Results are reported with Spearman correlation coefficients (*ρ*) values reported along with their 95% bootstrap confidence intervals.

#### 2.9.2. Image Processing and Data Analysis for Patients with PDAC

ROIs were manually delineated on DW and DCE images using ITK-SNAP software by an experienced radiologist, referencing anatomical images. DW and DCE image postprocessing, along with QIB map generation and the extraction of QIB values, were performed using MRI-QAMPER software, and the AIF was extracted from the abdominal aorta, as previously described using the automated AIF detection methods available in MRI-QAMPER software [16,39].

## 3. Results

### 3.1. Insights into the Architecture of the TME in PDAC

**Figure 2:**
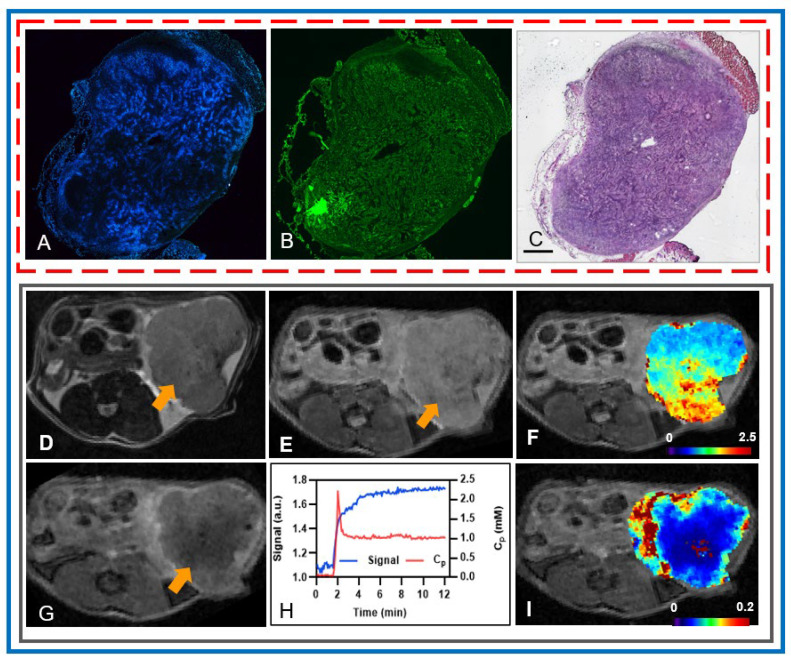
Top panel: Representative images of a PDAC tumor in an athymic mouse showing (**A**) perfused blood vessels, (**B**) abundant collagen fibers comprising a large portion of the tumor, and (**C**) H&E staining. Scale bar = 1 mm. Images (**A**,**C**) are from the same section; image B from an adjacent section (10 μm separation). Middle panel: (**D**) T_2_w image, (**E**) DW image (b = 0 s/mm^2^), and (**F**) ADC map (ADC, ×10^−3^ mm^2^/s). Bottom panel: (**G**) Postcontrast T_1_w image from the dynamic series (40th phase), (**H**) time course of the signal from the dynamic series along with the plasma CA concentration-time course (Cp) used for extended Tofts pharmacokinetic modeling, and (**I**) K^trans^ map (K^trans^, min^−1^). ADC and K^trans^ maps are overlaid on (**E**,**G**) images. Yellow arrows indicate PDAC tumor. Increased diffusivity, indicated by higher ADC values, is observed in regions with lower cell density, while elevated vascular permeability is evident at the tumor periphery.

Hoechst and H&E staining illustrate that the TME is dominated by dense desmoplastic stroma comprising pancreatic cancer–associated fibroblasts, a collagenous extracellular matrix (ECM), immune cells, and abnormal vasculature (Figure 2A–C). These findings also suggest that the preclinical PDAC model is matrix-rich and well-suited for the investigations described here. Histology of orthotopic tumors generated from this model in C57/Bl6 mice, as described by Pitter et al. [5], is visually very similar to the images presented here. Post-treatment mpMRI-derived QIBs, including ADC and K^trans^, also capture heterogeneity within the TME by reflecting variations in cellular density and vascular permeability. T2w (Figure 2D) and DW (Figure 2E) images demonstrate tumor morphology and with areas of restricted diffusion. On the ADC map, low and high ADC values correspond highly to less restricted diffusion (Figure 2F). Post-contrast T1w images exhibit the distribution of the CA within the tumor region and its enhancement (Figure 2G), and the plot shows the representative tumor tissue signal and the AIF used in the pharmacokinetic modeling (Figure 2H). In contrast, regions with elevated vascular permeability on the K^trans^ maps, particularly at the tumor periphery (Figure 2I), reflect increased microvascularity, contributing to greater CA extravasation.

**Figure 3:**
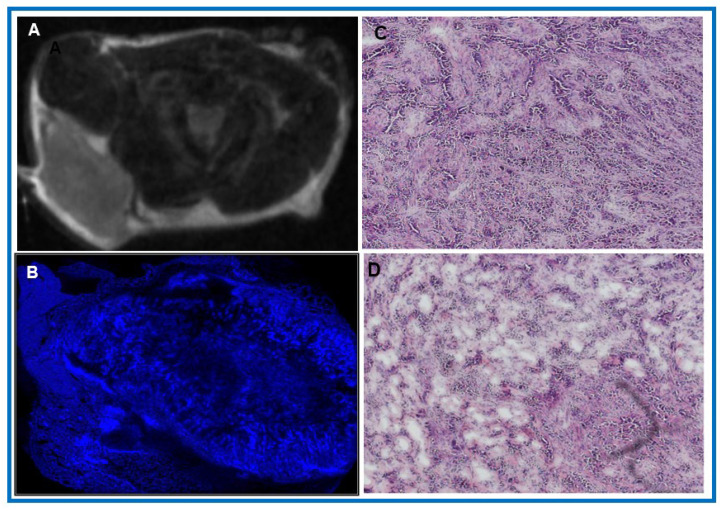
(**A**) Representative post-treatment T_2_w image of a PDAC tumor in an athymic mouse. (**B**) Hoechst 3342 staining, corresponding closely to matched histology images from the tumor, highlights regions of low or absent perfusion. (**C**,**D**) H&E-stained section, with nuclei in blue-purple, while eosin stains the cytoplasm and extracellular matrix pink. Stromal content was scored based on subjective visual assessment: Score 1: <50% stroma in the slice; Score 2: 51–100% stroma in the slice. Scale bar = 200 μm.

Figure 3A shows a representative post-treatment T_2_w image providing anatomical context by highlighting the differences in T_2_ relaxation times within the tumor and across surrounding tissue, while Hoechst staining reveals reduced perfusion in highly fibrotic regions and elevated perfusion in peripheral zones (Figure 3B). Spatial heterogeneity is a hallmark of PDAC and is largely shaped by the stroma, which constitutes 50–80% of the tumor volume. High-resolution H&E sections further illustrate areas of both sparse and dense stroma within a typical PDAC tumor (Figure 3C,D). Together, these images underscore how stromal composition drives spatial heterogeneity and perfusion dynamics in PDAC.

**Figure 4:**
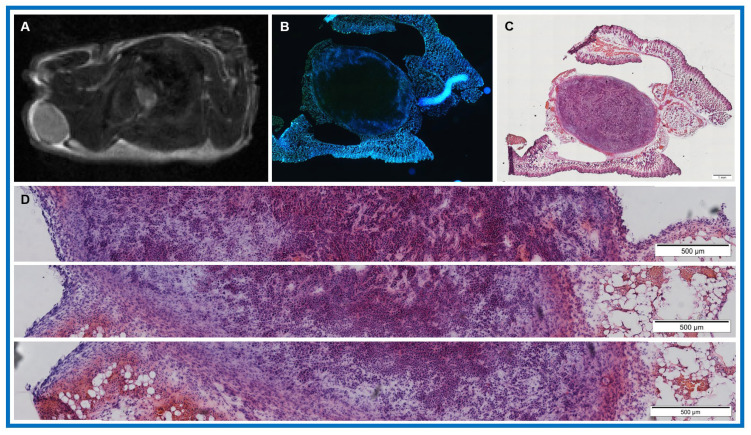
(**A**) Representative post-treatment T_2_w MR image of a PDAC tumor in an athymic mouse shows the tumor’s anatomical structure. (**B**) Hoechst 3342 staining highlights regions of active perfusion, predominantly localized to the tumor periphery, indicating heterogeneous vascular accessibility. (**C**) Corresponding H&E-stained section. (**D**) H&E tiles from three tumor regions analyzed using DeepLIIF. The total percentage of tumor cell in these tiles were 58.38%, 51.66%, and 49.21% (top to bottom), respectively, illustrating the regional heterogeneity in tumor composition and supporting the imaging-based assessment of the TME, respectively.

Figure 4 exhibits representative H&E-stained tiles from three tumor regions of a single slice analyzed from a PDAC tumor in an athymic mice using the DeepLIIF algorithm. The total numbers of tumor cells in the three tiles were 4534, 3594, and 3353, and the corresponding nuclei counts were 7766, 6957, and 6813. These values were obtained using the DeepLIIF algorithm, which employs a trained neural network to simultaneously generate tumor cell counts, nuclei count, and the percentage of tumor cells from standard H&E-stained images. Importantly, this approach enables high-throughput, quantitative assessment of TME directly from standard H&E images. The observed variation in tumor cell count across tiles reflects spatial heterogeneity, highlighting localized differences in tumor cellularity within the TME.

Table 1 further summarizes the DeepLIIF quantitative analysis of H&E-stained tissue samples from athymic mice (n = 10), illustrating the robustness of DeepLIIF for assessing tumor cells across H&E.

### 3.2. Potential of mpMRI-Derived QIBs to Determine Early Treatment Response

**Figure 5:**
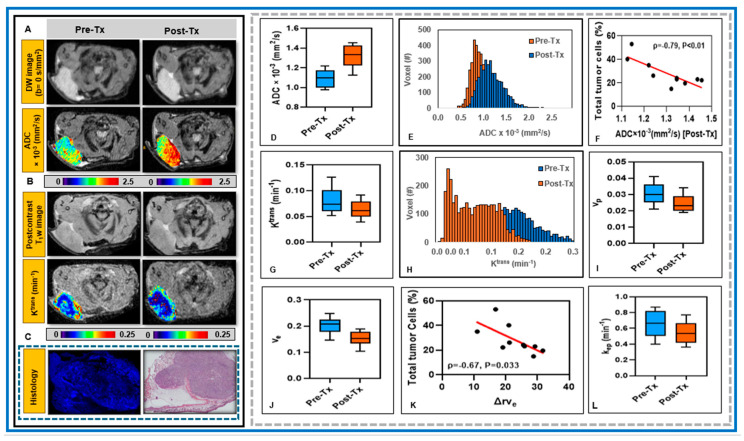
**Left Panel:** (**A**,**B**) Representative DW (b = 0 s/mm^2^) and Post contrast T_1_w images of a PDAC tumor in an athymic mouse, acquired at pre-treatment and after 10 Gy irradiation (post-treatment). (**A**) ADC and (**B**) K^trans^ maps overlaid on the DW (b = 0 s/mm^2^) images and the 30th phase of the DCE images, respectively. (**C**) Histological images of tumor sections stained with Hoechst 3342 and H&E. Right Panel: Box-and-whisker plots compare pre- vs. post-treatment values for (**D**) ADC, (**G**) K^trans^, (**I**) v_e_, (**J**) v_p_, and (**L**) k_ep_. Pre- and post-treatment ADC, K^trans^, v_e_, v_p_, and k_ep_ values were significantly different (*p* < 0.01). (**E**,**H**) Histogram plots show the pixel value distributions for ADC and K^trans^. (**F**,**K**) Spearman correlation plots demonstrate significant negative correlations between post-treatment ADC or Δrv_e_ and the total percentage of tumor cells (*p* < 0.05).

Changes in cellularity and blood flow are hallmarks of an early treatment response. Figure 5A,B shows representative pre- and post-treatment DW and T_1_w images as well as ADC and K^trans^ maps from the preclinical model of PDAC treated with radiotherapy. ADC and K^trans^ maps reveal increased water diffusivity and decreased vascular permeability, respectively, following tumor irradiation. Hoechst and H&E staining are from the section shown in Figure 5A and/or Figure 5B (Figure 5C).

Table 2 summarizes the changes in QIB values between pre- and post-treatment. Mean ADC values were significantly higher post-treatment than pre-treatment (*p* < 0.01; Figure 5D, Table 2), with a mean ΔrADC% of 20.50%. Mean values of K^trans^, v_e_, v_p_, and k_ep_ derived from the extended Tofts model were significantly lower post-treatment than pre-treatment (*p* < 0.05; Figure 5G,I,J,L; Table 2). Similarly, K^trans^ and v_p_ derived from the Patlak model showed significant reductions after tumor irradiation (Table 2). The mean relative changes were rΔK^trans^ = 20.41%, rΔv_e_ = 23.23%, rΔv_p_ = 17.93%, and rΔk_ep_ = 17.87% for the extended Tofts model, and ΔK^trans^ = 18.74% and rΔv_p_ = 23.78% for the Patlak model.

Figure 5F,K shows the scatter plot of post-treatment ADC and the relative change in v_e_ (rΔv_e_) values against the total percentage of tumor cells (%). Post-treatment ADC exhibited a strong inverse correlation with the total percentage of tumor cells (ADC: *ρ* = −0.77, *p* < 0.014). Similarly exhibited a strong correlation with the total percentage of tumor cells (*ρ* = −0.77, *p* = 0.009).

All parameters showed significant changes between pre- and post-treatment. ADC increased significantly (mean difference = 0.21, 95% CI [0.17–0.26], *p* < 0.01). For the extended Tofts model K^trans^ (−0.017, 95% CI [−0.023 to −0.011]), v_e_ (−0.05, 95% CI [−0.06, −0.039]), v_p_ (−0.006, 95% CI [−0.007, −0.004]), and k_ep_ (−0.12, 95% CI [−0.157, −0.081], as well as the Patlak model-estimated K^trans^ (−0.001, 95% CI [−0.002, −0.001]) and v_p_ (−0.011, 95% CI [−0.012, −0.01]), all QIBs values decreased significantly (*p* < 0.01).

Figure 5E,H shows the histogram plots for pixel value distributions of ADC and K^trans^ values within the tumor. The rightward shift in ADC indicates increased water diffusivity consistent with reduced cellularity, while the decrease in K^trans^ reflects reduced vascular permeability and perfusion following irradiation.

### 3.3. Feasibility of Obtaining QIBs from Patients with PDAC at Pre-Treatment mpMRI

Based on standard pre-treatment mpMRI in patients with PDAC, 3 tumors were found in the pancreatic head, 4 in the body, and 4 in the tail. The median tumor volume was 11.5 cm^3^ (range: 4.72–66.25 cm^3^) on DW MRI and 17.5 cm^3^ (range: 8.13–68.75 cm^3^) on DCE MRI. Mean ADC and K^trans^ from Patlak and extended Tofts models were 1.76 × 10^−3^ mm^2^/s, 0.095 min^−1,^ and 0.24 min^−1^, respectively (Table 3).

**Figure 6:**
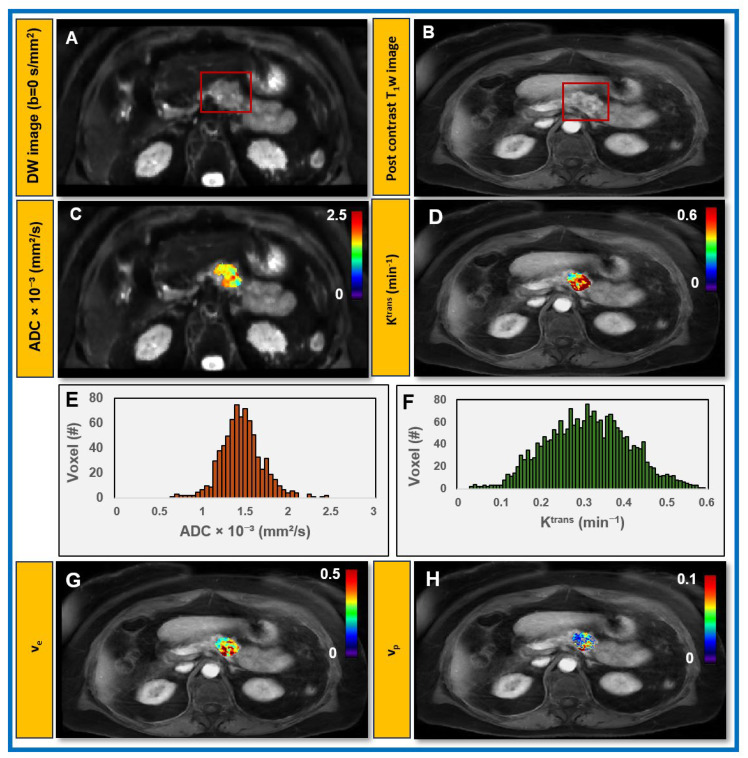
(**A**,**B**) Representative pre-treatment mpMR images and their derived parametric maps obtained from a female patient with PDAC (67 years) on clinical follow-up. (**C**) ADC map, (**D**) K^trans^, (**G**) v_e_, and (**H**) v_p_. Maps illustrating spatial heterogeneity within the tumor volume. Representative histograms of voxel-wise (**E**) ADC and (**F**) K^trans^ values within tumors highlight their distributions and heterogeneity, reflecting tumor cellularity as well as perfusion and vascular permeability. The tumor region is highlighted by the red rectangle.

Figure 6 shows representative ADC maps derived using a monoexponential fit of DW-MRI data, alongside extended Tofts model-derived maps of K^trans^, v_e_, and v_p_ from DCE data for patients with PDAC at pre-treatment. These maps illustrate the spatial heterogeneity within the tumor volume. Representative histograms of ADC and K^trans^ values, generated from voxel-wise data for a patient with PDAC, highlight the distribution and heterogeneity of these QIBs within the tumor, providing insight into TME characteristics such as cellular density and vascular permeability.

## 4. Discussion

This study establishes a foundation for future co-clinical research to evaluate emerging drug combinations targeting both the tumor and stroma by advancing our understanding of the TME in PDAC. The TME is characterized by heterogenous cellular composition, aberrant vascularity, as well as diverse immune and stromal components [34]. Spatial variation in stromal composition influences both water diffusion and perfusion dynamics, thereby hampering drug delivery and therapeutic efficacy. Thus, understanding the interactions within the TME between ECM components and tumor cells, which govern tumor growth and metastasis, is critical for developing novel drugs and strategies. In this context, mpMRI-derived QIBs have shown promise in providing insights into the TME for treatment assessment and drug delivery [48,49,50,51,52,53].

Our preclinical model of PDAC treated with radiotherapy shows that the correlation of early post-treatment mpMRI-derived QIBs (ADC, K^trans^, v_e_, and v_p_) with histology-based cell counts provides a robust approach for assessing early post-treatment changes in the TME. Irradiation led to measurable changes in the ADC (~20.0%) and vascular-related parameters (K^trans^, v_e_, v_p_, k_ep_) of up to 23%, indicating reduced cellularity and radiation-induced vascular damage. Hoechst staining distinguished well-perfused from poorly perfused regions, while DeepLIIF revealed substantial cellular variability (15–53%). mpMRI-derived QIBs also proved valuable for characterizing PDAC in patients, with ADC and K^trans^ histograms capturing spatial intratumoral heterogeneity. Treatment induced a significant increase in ADC values, suggesting reduced tumor cell density and expanded extracellular space, whereas K^trans^, v_e_, and v_p_ decreased, reflecting reduced vascular perfusion/permeability, CA distribution space in the vascular space and EES. The effect sizes for all parameters were large (r > 0.5), indicating that these changes were not only statistically significant but also substantial and likely biologically meaningful [54], reflecting notable alterations in tumor cellularity, tissue microstructure, and the microvascular integrity.

Dense tumor cells restrict water diffusion, resulting in lower ADC values, whereas tumors with abundant stroma are less cellular, resulting in higher ADC values [22]. Heid et al., in a co-clinical assessment of tumor cellularity in PDAC, demonstrated a significant negative correlation between ADC values and percentages of tumor cells [15]. Further, they reported an inverse relationship between tumor cellularity and stroma content. Thus, ADC provides an indirect measure of tumor cellularity and stromal content, offering potential value in tumor characterization and treatment response assessment. KRAS inhibitors show therapeutic benefit in PDAC, but resistance limits their efficacy [55]. Gupta et al. reported that a negative correlation between the percentage change in ADC values and tumor cellularity, as estimated from H&E-stained tumor sections following KRASi treatment [56], suggesting that increased ADC is indicative of reduced cellularity in responding PDAC tumors [35]. In preclinical PDAC models, Romanelli et al. reported that ADC has shown correlation with histological features such as necrosis and stromal density [57]. Consistent with these previous results, in our study, pre- and post-treatment ADC exhibited a significant negative correlation with the total percentage of tumor cells obtained from H&E images using DeepLIIF. Effective therapy reduces the densely packed cells and collagen that impede water diffusion, reflected in a higher ADC value [58]. Changes in longitudinal ADC values have been significantly correlated with treatment response in PDAC patients undergoing chemoradiation therapy [16,26]. While our study and those studies discussed above focus on the ADC metric, the DKI-derived diffusion metric has been reported to be highly negatively correlated with the percentage of tumor and stroma seen in histology H&E, underscoring the potential of DKI also for both tumor characterization and therapeutic response assessment in PDAC [32].

Beyond water diffusion metrics, quantitative modeling of tissue CA concentration, using pharmacokinetic models, enables TME characterization through metrics capturing blood flow, vascular permeability, and CA leakage space [59]. In this study, we applied two complementary models: the Patlak model, suitable for low temporal resolution data and tissues with minimal leakage and irreversible exchange, and the extended Tofts model, which accounts for reversible exchange. Both models assume the fast exchange limit for modeling of the tissue CA concentration and K^trans^, which reflects both perfusion and permeability. Analyses of these models help identify the key physiological processes influenced by the desmoplastic stroma and interstitial fluid pressure in PDAC. Wegener et al. found that differentiated tumors had lower collagen I and IV, and that ADC and v_e_ effectively distinguished collagen-rich non-differentiated tumors from collagen-poor differentiated ones [10]. Romanelli reported that the K^trans^ maps revealed distinct perfusion and permeability patterns in KPC versus CKS tumors, consistent with CD31 staining [57]. Despite increased neovascularization in KPC tumors, their lower K^trans^ values likely result from dense ECM components such as collagen and hyaluronan, which elevate interstitial fluid pressure and hinder effective perfusion. Cao et al. reported that the K^trans^ can identify early responses to stroma-targeted therapy of PEGPH20 combined with gemcitabine [13]. Interpreting K^trans^ in the context of radiotherapy as applied in our study requires understanding the blood flow and permeability. In PDAC, where vasculature is often compromised, radiotherapy may further reduce perfusion, resulting in lower K^trans^ and v_p_ values. Fukukura et al. reported that higher v_e_ was associated with chemotherapy response, reflecting stromal and cellular changes [20]. Changes in rΔv_e_ (~23%) values post-radiotherapy may reflect alterations in cancer-associated fibroblasts and ECM remodeling [60]. In our preclinical model, following 10 Gy irradiation, QIB values decreased, reflecting radiation-induced vascular damage and reduced vascular permeability and the leakage space, underscoring the importance of monitoring microvascular and stromal dynamics during therapy. However, the selection of the pharmacokinetic model and AIF used in the analysis can influence the estimates of QIBs, making their standardization challenging [61].

In the era of targeted therapies, QIBs have shown promise for longitudinal assessment of therapeutic effects, often detecting changes in tumor cellularity and vascularization before morphologic alterations [51,62]. These early insights are particularly valuable in PDAC, where dense stroma hampers drug delivery and treatment response. In preclinical PDAC models, PEGPH20, a stromal-targeted agent, has demonstrated the ability to deplete hyaluronan, reduce interstitial fluid pressure, and enhance drug delivery. PEGPH20 treatment was associated with increased DCE-MRI parameters (K^trans^, v_e_, and v_p_), consistent with stromal HA depletion, reduced interstitial pressure, and improved perfusion and vascular space [24]. Arias-Lorza et al. analyzed results from early-phase clinical trials and confirmed these imaging findings, showing increased K^trans^, v_e_, and v_e_ as early as day 1 post-treatment [24]. However, phase III trial results in patients with hyaluronan-high metastatic PDAC revealed that while PEGPH20 improved objective response rates when combined with nab-paclitaxel and gemcitabine, overall survival and progression-free survival rates did not improve [63]. These results suggest that stromal remodeling alone may be insufficient and highlight the need for better patient selection strategies and combination approaches. Recent advances in KRAS-targeted therapies have further underscored the potential of QIBs. Gupta et al. reported that K^trans^ was inversely associated with CD31 for tumor microvascular density [56]. These findings emphasize the importance of integrating molecular profiling with imaging biomarkers to guide therapy. Together, these studies demonstrate that QIBs like K^trans^ can serve as a biomarker for early response assessment in PDAC.

This study highlights the pivotal role of quantitative imaging biomarkers (QIBs) derived from multiparametric MRI (mpMRI) in characterizing pancreatic ductal adenocarcinoma (PDAC) in both preclinical and clinical studies. mpMRI-based QIBs, such as ADC, K^trans^, and v_p_, enable noninvasive assessment of tumor physiology and TME heterogeneity. These capabilities are especially critical in PDAC, where conventional imaging often fails to capture early biological changes due to the tumor’s dense stromal architecture. Importantly, recent clinical trials have demonstrated that changes in QIBs can precede morphologic response as well as correlate with therapeutic outcomes, particularly in studies involving PEGPH20 and KRAS-targeted agents [13,56]. The ability to detect stromal remodeling and vascular changes within days of treatment initiation highlights the translational potential of these QIBs.

There are a few limitations in the study. First, the treatment modalities differed between the preclinical and clinical cohorts: Radiotherapy was assessed in the preclinical PDAC model involving mice, while patients received systemic chemotherapy (mFOLFIRINOX) and pancreatic enzyme replacement therapy. However, the primary aim of this study was not to compare treatment responses directly, but to investigate the TME using mpMRI-derived QIBs in PDAC and to explore the feasibility of obtaining QIBs in PDAC patients using clinically approved MRI sequences. Second, both cohorts had small sample sizes, which limits the generalizability of the findings, which need validation in larger cohort studies. Third, MRI-derived QIBs and histologic features were correlated at the entire tumor level and not at individual slices. Future studies are needed to validate these initial results and support clinical translation.

## 5. Conclusions

This study demonstrates the potential of mpMRI-derived QIBs for assessing the TME in PDAC, capturing early changes in tumor cellularity and vascular function in response to therapy. DeepLIIF enabled reproducible, scalable assessment of tumor cell count from H&E. This study establishes a foundation for co-clinical research to evaluate emerging drug combinations targeting both tumor and stroma, advancing our understanding of the TME in PDAC.

## Figures and Tables

**Table 1:** Summary of DeepLIIF quantitative analysis of H&E-stained tissue samples from the preclinical PDAC model.

Value	Total Tumor Cells (/μm^2^)	Total Nuclei	Total Percentage of Tumor Cells (%)
Median (min, max)	3408 (1403, 20,629)	8650 (4696, 118,138)	24 (15, 53)
Mean ± SD	6633.0 ± 6867.0	32,283.0 ± 40,211.0	28.0 ± 11.0

**Table 2:** Summary of pre- and post-treatment DW and DCE MRI data modeling and model-derived QIB values in a preclinical model of PDAC treated with radiotherapy.

Model	Parameter	Values		|ΔrX (%)|(Unitless)
		Pre-Treatment	Post-Treatment	
Monoexponential	ADC × 10^−3^ (mm^2^/s)	1.10 ± 0.09	1.31 ± 0.10 *	20.50 ± 7.37
Patlak Model	K^trans^ (min^−1^)	0.007 ± 0.003	0.0057 ± 0.002 *	18.74 ± 7.4
	v_p_	0.0460 ± 0.004	0.0350 ± 0.003 *	23.78 ± 3.22
Extended Tofts Model	K^trans^ (min^−1^)	0.080 ± 0.025	0.063 ± 0.017 *	20.41 ± 5.24
	v_e_	0.20 ± 0.03	0.16 ± 0.030 *	23.23 ± 6.10
	v_p_	0.030 ± 0.007	0.025 ± 0.005 *	17.93 ± 5.52
	k_ep_ (min^−1^)	0.66 ± 0.16	0.54 ± 0.14 *	17.87± 7.87

* WSRT (*p* < 0.01).

**Table 3:** Summary of Pre-treatment DW- and DCE-MRI data modeling and model-derived QIB values in patients with PDAC.

Model	Parameter (Units)	Values
Monoexponential	ADC (×10^−3^ mm^2^/s)	1.76 ± 0.0.56
Patlak Model	K^trans^ (min^−1^)	0.095 ± 0.053
	v**_p_**	0.067 ± 0.039
Extended Tofts Model	K^trans^ (min^−1^)	0.24 ± 0.12
	v_e_	0.36 ± 0.11
	v_p_	0.043 ± 0.029
	k_ep_ (min^−1^)	0.70 ± 0.20

## Data Availability

The data presented in this study will be provided upon reasonable request.

## Abbreviations

The following abbreviations are used in this manuscript:

ADC	Apparent diffusion coefficient
AIF	Arterial input function
CA	Contrast agent
DISCO	Differential Subsampling with Cartesian ordering
DKI	Diffusion kurtosis imaging
DW	Diffusion-weighted
DCE	Dynamic contrast-enhanced
ECM	Extracellular matrix
EES	Extravascular extracellular space
FA	Flip angle
FLASH	Fast Low Angle Shot
FOV	Field of view
H&E	Hematoxylin and eosin
LAVA	Liver Acquisition with Volume Acceleration
mpMRI	Multiparametric magnetic resonance imaging
MS	Matrix size
NA	Number of averages
NS	Number of slices
PDAC	Pancreatic ductal adenocarcinoma
PERT	Pancreatic enzyme replacement therapy
PET	Positron emission tomography
RARE	Rapid Acquisition with Relaxation Enhancement
rFOV	Reduced field of view
ROI	Region of interest
STAR	Stack-of-stars
TE	Echo time
TME	Tumor microenvironment
TR	Repetition time
QIB	Quantitative imaging biomarker
